# How Evidence-Based Are the Recommendations in Evidence-Based Guidelines?

**DOI:** 10.1371/journal.pmed.0040250

**Published:** 2007-08-07

**Authors:** Finlay A McAlister, Sean van Diepen, Rajdeep S Padwal, Jeffrey A Johnson, Sumit R Majumdar

**Affiliations:** Italian Cochrane Centre, Italy; 1 The Division of General Internal Medicine, University of Alberta, Edmonton, Canada; 2 The Institute of Health Economics, University of Alberta, Edmonton, Canada; 3 The School of Public Health, University of Alberta, Edmonton, Canada

## Abstract

**Background:**

Treatment recommendations for the same condition from different guideline bodies often disagree, even when the same randomized controlled trial (RCT) evidence is cited. Guideline appraisal tools focus on methodology and quality of reporting, but not on the nature of the supporting evidence. This study was done to evaluate the quality of the evidence (based on consideration of its internal validity, clinical relevance, and applicability) underlying therapy recommendations in evidence-based clinical practice guidelines.

**Methods and Findings:**

A cross-sectional analysis of cardiovascular risk management recommendations was performed for three different conditions (diabetes mellitus, dyslipidemia, and hypertension) from three pan-national guideline panels (from the United States, Canada, and Europe). Of the 338 treatment recommendations in these nine guidelines, 231 (68%) cited RCT evidence but only 105 (45%) of these RCT-based recommendations were based on high-quality evidence. RCT-based evidence was downgraded most often because of reservations about the applicability of the RCT to the populations specified in the guideline recommendation (64/126 cases, 51%) or because the RCT reported surrogate outcomes (59/126 cases, 47%).

**Conclusions:**

The results of internally valid RCTs may not be applicable to the populations, interventions, or outcomes specified in a guideline recommendation and therefore should not always be assumed to provide high-quality evidence for therapy recommendations.

## Abstract 2

From an analysis of cardiovascular risk-management recommendations in guidelines produced by pan-national panels, McAlister and colleagues concluded that fewer than half were based on high-quality evidence.

## Editors' Summary

**Background.:**

Until recently, doctors largely relied on their own experience to choose the best treatment for their patients. Faced with a patient with high blood pressure (hypertension), for example, the doctor had to decide whether to recommend lifestyle changes or to prescribe drugs to reduce the blood pressure. If he or she chose the latter, he or she then had to decide which drug to prescribe, set a target blood pressure, and decide how long to wait before changing the prescription if this target was not reached. But, over the past decade, numerous clinical practice guidelines have been produced by governmental bodies and medical associations to help doctors make treatment decisions like these. For each guideline, experts have searched the medical literature for the current evidence about the diagnosis and treatment of a disease, evaluated the quality of that evidence, and then made recommendations based on the best evidence available.

**Why Was This Study Done?:**

The recommendations made in different clinical practice guidelines vary, in part because they are based on evidence of varying quality. To help clinicians decide which recommendations to follow, some guidelines indicate the strength of their recommendations by grading them, based on the methods used to collect the underlying evidence. Thus, a randomized clinical trial (RCT)—one in which patients are randomly allocated to different treatments without the patient or clinician knowing the allocation—provides higher-quality evidence than a nonrandomized trial. Similarly, internally valid trials—in which the differences between patient groups are solely due to their different treatments and not to other aspects of the trial—provide high-quality evidence. However, grading schemes rarely consider the size of studies and whether they have focused on clinical or so-called “surrogate” measures. (For example, an RCT of a treatment to reduce heart or circulation [“cardiovascular”] problems caused by high blood pressure might have death rate as a clinical measure; a surrogate endpoint would be blood pressure reduction.) Most guidelines also do not consider how generalizable (applicable) the results of a trial are to the populations, interventions, and outcomes specified in the guideline recommendation. In this study, the researchers have investigated the quality of the evidence underlying recommendations for cardiovascular risk management in nine evidence-based clinical practice guides using these additional criteria.

**What Did the Researchers Do and Find?:**

The researchers extracted the recommendations for managing cardiovascular risk from the current US, Canadian, and European guidelines for the management of diabetes, abnormal blood lipid levels (dyslipidemia), and hypertension. They graded the quality of evidence for each recommendation using the Canadian Hypertension Education Program (CHEP) grading scheme, which considers the type of study, its internal validity, its clinical relevance, and how generally applicable the evidence is considered to be. Of 338 evidence-based recommendations, two-thirds were based on evidence collected in internally valid RCTs, but only half of these RCT-based recommendations were based on high-quality evidence. The evidence underlying 64 of the guideline recommendations failed to achieve a high CHEP grade because the RCT data were collected in a population of people with different characteristics to those covered by the guideline. For example, a recommendation to use spironolactone to reduce blood pressure in people with hypertension was based on an RCT in which the participants initially had congestive heart failure with normal blood pressure. Another 59 recommendations were downgraded because they were based on evidence from RCTs that had not focused on clinical measures of effectiveness.

**What Do These Findings Mean?:**

These findings indicate that although most of the recommendations for cardiovascular risk management therapies in the selected guidelines were based on evidence collected in internally valid RCTs, less than one-third were based on high-quality evidence applicable to the populations, treatments, and outcomes specified in guideline recommendations. A limitation of this study is that it analyzed a subset of recommendations in only a few guidelines. Nevertheless, the findings serve to warn clinicians that evidence-based guidelines are not necessarily based on high-quality evidence. In addition, they emphasize the need to make the evidence base underlying guideline recommendations more transparent by using an extended grading system like the CHEP scheme. If this were done, the researchers suggest, it would help clinicians apply guideline recommendations appropriately to their individual patients.

**Additional Information.:**

Please access these Web sites via the online version of this summary at http://dx.doi.org/10.1371/journal.pmed.0040250.

• Wikipedia contains pages on evidence-based medicine and on clinical practice guidelines (note: Wikipedia is a free online encyclopedia that anyone can edit; available in several languages)

• The National Guideline Clearinghouse provides information on US national guidelines

• The Guidelines International Network promotes the systematic development and application of clinical practice guidelines

• Information is available on the Canadian Hypertension Education Program (CHEP) (in French and English)

• See information on the Grading of Recommendations Assessment, Development and Evaluation (GRADE) working group, an organization that has developed an grading scheme similar to the CHEP scheme (in English, Spanish, French, German, and Italian)

## Introduction

There has been a rapid expansion in the number of clinical practice guidelines over the past decade and, as a result, clinicians are frequently faced with several guidelines for treatment of the same condition. Unfortunately, recommendations may differ between guidelines [1,2], leaving the clinician with a decision to make about which guideline to follow. While it is easy to say that one should follow only those guidelines that are “evidence based,” very few guideline developers declare their documents to be non–evidence based, and there is ambiguity about what “evidence based” really means in the context of guidelines. The term may be interpreted differently depending on who is referring to the guideline—the developer, who creates the guidelines, or the clinician, who uses them. To their developers, “evidence-based guidelines” are defined as those that incorporate a systematic search for evidence, explicitly evaluate the quality of that evidence, and then espouse recommendations based on the best available evidence, even when that evidence is not high quality [3]. However, to clinicians, “evidence based” is frequently misinterpreted as meaning that the recommendations are based solely on high-quality evidence (i.e., randomized clinical trials [RCTs]) [4]. Previous studies of guidelines have focused almost exclusively on the elements embodied in the first definition of an evidence-based guideline. For example, guideline appraisal tools assess the methodology used in developing the guideline and the clarity with which recommendations and the type of underlying evidence are communicated in that guideline [5,6].

However, few studies have addressed the issue raised in the second interpretation of an evidence-based guideline—that is, the quality of the evidence underpinning “evidence-based” guidelines. Given the widespread availability of electronic databases to search the literature, one would expect that evidence-based guidelines would usually cite the same evidence. However, an analysis of 15 guidelines for type 2 diabetes mellitus revealed little overlap—only ten studies (less than 1% of all citations) were cited in at least six of these guidelines, and the most frequently cited study in these guidelines (the Diabetes Complication Control Trial, referenced in 11 of 15 guidelines) was conducted exclusively in patients without type 2 diabetes mellitus [7].

How then should the quality of evidence underlying recommendations be evaluated? In those guidelines that use explicit scales, virtually all are based solely on considerations of study design and internal validity [8]. For example, RCTs are graded higher than observational studies irrespective of sample size, study conduct, endpoints evaluated, or the applicability (i.e., generalizability) of the RCT to the populations, interventions, and outcomes specified in the guideline recommendation. In order to incorporate external validity, applicability, and clinical relevance into evidence appraisal, we used an evidence grading scheme that has been used (and refined) for almost a decade by the Canadian Hypertension Education Program (CHEP) [9]. The CHEP scheme evaluates three (type of study, internal validity, and directness) of the four domains recommended by the Grading of Recommendations Assessment, Development and Evaluation [GRADE] working group (http://www.gradeworkinggroup.org/, last accessed 29 January 2007)—consistency between studies is not explicitly evaluated in the CHEP scheme (except in the assessment of meta-analyses) and, instead, CHEP places primacy on the study achieving the highest evidence grading for a particular recommendation [3].

We designed this study to evaluate the quality of the evidence cited for cardiovascular risk management recommendations in evidence-based clinical practice guidelines.

## Methods

### Selection of Guidelines

Based on the prevalence of these conditions in the outpatient primary care setting and our collective areas of clinical expertise, we restricted ourselves to national guidelines for the management of diabetes mellitus, dyslipidemia, and hypertension. We a priori chose the most recent guidelines from the United States, Canada, and Europe for each disorder, as these are the national-level guidelines Canadian clinicians are most commonly exposed to [10–19]. We defined recommendations as any statements that advocated a specific intervention for application in clinical care. We focused on evaluating the evidence base for cardiovascular risk management interventions in these guidelines, and did not examine the evidence base underlying recommendations on diagnosis, monitoring, or prevention. Moreover, as our interest was on the chronic treatment of these conditions, we a priori excluded recommendations for pregnant, hospitalized, or peri-operative individuals.

### Quality Appraisal of the Guidelines

We appraised the quality of each guideline using the standardized Appraisal of Guidelines Research and Evaluation (AGREE) instrument (http://www.agreetrust.org/, last accessed 29 January 2007), which consists of 23 items that rate the various dimensions of quality for the overall guideline using four-point Likert scales. The AGREE instrument is organized into six independent domains, and the score for each domain is calculated as a percentage of the maximum possible score for that domain [20]. Three investigators independently completed the AGREE instrument for each guideline, and discrepancies were resolved by consensus.

It should be noted that the AGREE appraisal is based on consideration of the whole guideline (i.e., not for specific recommendations within each guideline) and includes any recommendations on diagnosis, monitoring, or prevention. In contrast, our detailed analysis of the evidence underlying recommendations focused solely on therapy recommendations.

### Extraction of Recommendations and Underlying Evidence

After completing a training set to calibrate evidence ratings, two investigators with expertise in both critical appraisal and the topic areas of the chosen guidelines reviewed each guideline, independently extracted recommendations and the references cited by each guideline in support of each cardiovascular therapy recommendation, and graded the quality of the evidence as described in detail below. We did not conduct literature searches and used only the studies cited directly in the references list of each guideline to evaluate the evidence base for that recommendation. All evidence ratings for each cardiovascular therapy recommendation were independently checked by a third reviewer (FAM), and any discrepancies were resolved by consensus.

### Grading Quality of the Underlying Evidence

We appraised the quality of evidence cited to support each cardiovascular risk management recommendation using the CHEP evidence-grading scheme (Figure 1). The CHEP grading scheme was developed in 1999, has been used and refined in the seven annual updates of the Canadian hypertension guidelines since then, and explicitly operationalizes many elements of the GRADE scheme using a priori standardized rules of evidence (based on reference [21]) in a fashion that has been shown to be reproducible within and between reviewers trained in critical appraisal [9]. As we were interested in exploring how many cardiovascular therapy recommendations considered to be high quality in current guidelines would have their evidence grading reduced when factors beyond study design were taken into account, we focused specifically on those recommendations citing RCTs or systematic reviews of RCTs. In situations where several RCTs or systematic reviews were cited in support of a particular recommendation, we based our evidence grade on the RCT or systematic review achieving the highest ranking for that recommendation.

**Figure 1:**
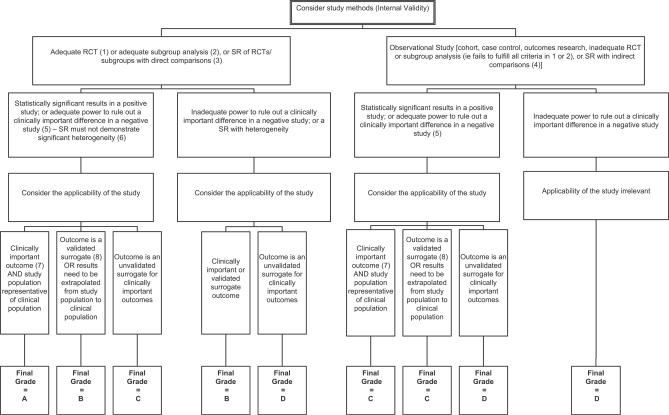
The Evidence-Grading Scheme Employed in this Study Adapted from CHEP [9]. An “adequate” RCT is one with allocation concealment, blinded assessment of outcomes (if subjective), intention-to-treat analysis, adequate follow-up (i.e., at least 90%, or losses to follow-up are too few to materially affect the results), and sufficient sample size to detect a clinically important difference with power > 80% (1). Subgroup analysis was a priori, done within an adequate RCT, one of only a few tested, and there was sufficient sample size within the examined subgroup to detect a clinically important difference with power > 80% (2). A sytematic review (SR) with direct comparisons is one in which the comparison arms are derived from head-to-head comparisons within the same RCT (3). A systematic review with indirect comparisons is one in which the comparison arms are derived from different RCTs, then extrapolations are made across RCTs (4). Adequate power in a negative study implies that the 95% confidence interval excludes a clinically important difference (5). Effect estimates in each study included in the systematic review are qualitatively similar (i.e., in the same direction) (6). Clinically important outcomes are “hard” endpoints such as death, stroke, or myocardial infarction (7). End points have been consistently shown to be associated with the clinical end point in multiple studies (observational or RCT), and RCTs have consistently demonstrated that improvement in the surrogate translates into a consistent and predictable improvement in the clinical end point (8).

Pooled across all nine guidelines, inter-rater agreement on whether a recommendation was based on RCT evidence or not was 91% (kappa 0.80) and inter-rater agreement on whether RCT evidence was high quality or not (grade A versus B, C, or D on the CHEP scheme) was 89% (kappa 0.78).

## Results

### Characteristics of the Guidelines and Recommendations

The nine guidelines in our study (Table 1) [10–19] ranged in size from ten pages to 284 pages and cited between 44 and 1,121 references. They provided a total of 1,005 recommendations for diagnosis and prevention (*n* = 362) or treatment (*n* = 643) for patients with diabetes mellitus, dyslipidemia, or hypertension; 369 of the treatment recommendations in these guidelines advocated cardiovascular risk management therapies. Although only four of the guidelines [10,11,14,17] provided grades for their underlying evidence, seven guidelines explicitly linked recommendations to evidence (either within the text of the recommendation itself or at the end of relevant sections of the guideline text); these 338 recommendations formed the sample for this study.

**Table 1:**
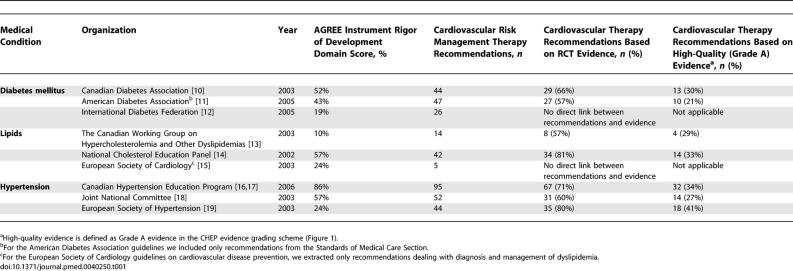
Description of Guidelines and Quality of Evidence

### Quality of the Guidelines

The standardized scores for each domain of the AGREE instrument (averaged across guidelines within the same topic areas) are outlined in Figure 2. While these guidelines were scored highly for the clarity of their presentations and their descriptions of “scope and purpose” and were judged to have reasonable levels of editorial independence, they scored poorly on the “stakeholder involvement” (due particularly to lack of involvement of patient groups and prepiloting of the guidelines) and “applicability” domains of the AGREE instrument. Within the applicability domain, these guidelines infrequently discussed resource implications, audit criteria, or potential organizational changes necessary to implement recommended therapies.

**Figure 2:**
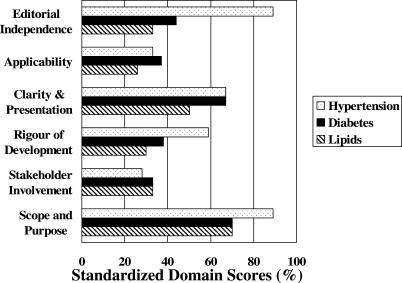
Summary of AGREE Domain Scores for Guidelines, Averaged over Each Condition

### Quality of the Underlying Evidence

Of the 338 recommendations that endorsed particular cardiovascular risk management therapies and cited evidence, 231 (68%) cited an RCT or systematic review of RCTs in support of that recommendation (Table 1). Of these 231 RCT-based recommendations, 105 (45%) received a grade A using the CHEP grading scheme (corresponding to “high-quality evidence” in the GRADE scheme), with the proportion ranging between 37% and 51% in different guidelines (Figure 3). Thus, only 28% (range 21% to 41% between guidelines) of the 369 cardiovascular risk management recommendations in these nine prominent national evidence-based guidelines were directly supported by high-quality evidence (Table 1).

**Figure 3:**
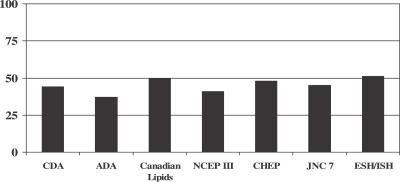
Percentage of Recommendations Citing RCTs That Were Based on High-Quality Evidence in Each Guideline Legend and references: ADA, American Diabetes Association [11]; Canadian Lipids, the Canadian Working Group on Hypercholesterolemia and Other Dyslipidemias [13]; CDA, Canadian Diabetes Association [10]; CHEP, the Canadian Hypertension Education Program [16,17]; ESH/ISH, European Society of Hypertension/International Society of Hypertension [19]; JNC 7, Joint National Committee seventh report [18]; NCEP III, Third Report of the National Cholesterol Education Program [14].

The most frequent reason for downgrading RCT-based therapy recommendations (64 [51%] of the 126 cases) were concerns about the need to extrapolate from a highly selected RCT population to the scenario and/or the target population specified in the guideline. As an illustration, consider the differences in quality of evidence underlying two antihypertensive recommendations that were both based on RCT evidence. The first recommendation was for the use of thiazides in individuals with hypertension. It cited a meta-analysis [22] of 30 RCTs that proved conclusively the efficacy of thiazides in reducing cardiovascular events and mortality in over 70,000 individuals with hypertension—this recommendation was judged to be supported by high-quality (grade A) evidence. In contrast, the second recommendation was for the use of spironolactone for individuals with hypertension and cited an RCT [23] of 1,663 patients with advanced heart failure in which baseline blood pressures were normotensive (mean 122/75 mm Hg), and results for the hypertensive subgroup were not reported separately. Although this spironolactone recommendation was based on RCT evidence, its relevance and applicability to the general hypertensive population is sufficiently uncertain that we classified it as grade B (moderate quality) evidence at best. As an aside, it should be noted that RCT evidence was not downgraded if the RCT was conducted primarily in men but the recommendation referred to people in general, or if the RCT was conducted in a country other than that from which the guideline arose. The second most common reason for downgrading RCT-based recommendations (59 cases, 47%) were concerns about the clinical relevance of the RCT—for example, the RCT reported the effect of the recommended therapy on surrogate outcomes only (e.g., levels of glucose, low-density lipoprotein cholesterol, or blood pressure) rather than patient-centered outcomes such as death, myocardial infarction, or stroke. Illustrative examples of RCT-based recommendations that were down-graded are provided in Table 2.

**Table 2:**
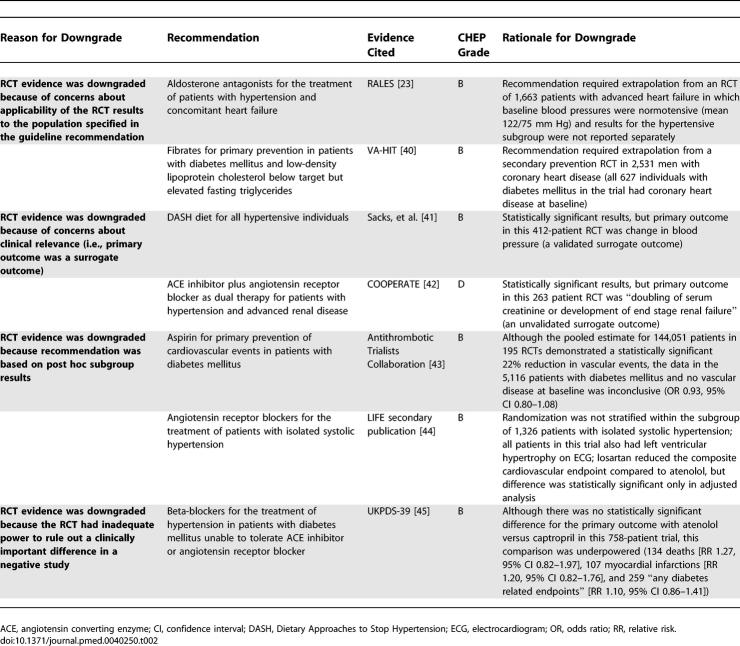
Examples of Guideline Recommendations That Cited an Internally Valid Randomized Trial Which Did Not Provide High-Quality Evidence for That Recommendation

The quality of the evidence base cited for cardiovascular risk management therapies was not related to guideline length (Pearson correlation coefficient *[r]* = 0.13, *p* = 0.78), number of references (*r* = 0.20, *p* = 0.67), or score on the “rigor of development” domain of the AGREE instrument (*r* = −0.03, *p* = 0.96).

## Discussion

In summary, we found that while two-thirds of cardiovascular risk management therapy recommendations made in the nine different guidelines we examined were based on RCT evidence, less than half of these RCT-based recommendations were deemed “high quality” using an evidence-grading scheme that went beyond considerations of internal validity alone to take into account clinical relevance and direct applicability of the RCT to that recommendation. As a result, less than one-third of recommendations that advocated specific cardiovascular risk management therapies in these evidence-based guidelines were actually based on high-quality evidence.

The most frequent reason for RCT-based recommendations to be down-graded was that the RCT was conducted to answer a particular question in a restricted study population but was then extrapolated in the guideline to justify using the tested intervention in a related, but different, clinical scenario and/or in a more general population. In a similar vein, other investigators have recently questioned whether the evidence cited in the Third Report of the National Cholesterol Education Program, or NCEP III, as support for recommendations to use statins for primary prevention of cardiovascular disease is directly applicable, since one-tenth of the patients in the 16 “primary prevention” trials cited in that guideline had cerebrovascular or peripheral vascular disease at baseline [24].

As a corollary, it is evident that while a particular RCT may be used as the basis for multiple recommendations, RCTs will not provide the same quality of evidence for each recommendation (and in some cases guideline developers may extrapolate beyond the limits of the evidence in making particular recommendations). For example, the 2003 Kidney Disease Outcome Quality Initiative guidelines [25] recommended statins for all patients with chronic kidney disease and LDL > 2.59 mmol/l, including those with end-stage renal disease, on the basis of RCTs such as the Heart Protection Study which were positive, but excluded patients with end-stage renal disease [26]. However, a recently published RCT conducted in 1255 hemodialysis patients with type 2 diabetes mellitus found no reduction in the primary outcome of cardiovascular events or death but instead an unexpected increase in the risk of stroke with statin therapy [27].

We do not mean to imply that recommendations should not be made in the absence of high quality evidence or that RCT evidence should not be extrapolated beyond the limits of trial eligibility criteria. Indeed, we recognize that trialists design RCTs with relatively homogenous populations in order to maximize internal validity (at the expense of external validity), and there are published guides on how and when to extrapolate RCT evidence to individual patient situations [28]. However, we do believe that transparency about any extrapolation of RCT evidence is critical, particularly in light of studies demonstrating that the composition and interpersonal dynamics of a guideline panel influence the extent to which their consensus recommendations diverge from the available evidence base [29–31].

Our findings that only some guidelines linked their recommendations to citations and that only some used explicit grading systems to communicate the quality of the evidence echo earlier reviews [7,32–34]. Similarly, our finding that many treatment recommendations are not based on RCT evidence has been reported before in other fields [35]. However, our unique finding is that even those recommendations in evidence-based guidelines that cite internally valid RCTs as support may not be underpinned by high-quality evidence. We did not compare the countries of origin for cited studies, because previous studies have already established that local evidence tends to be over-represented in guidelines [7,36,37]. While the guidelines we studied were published at different times, the range was narrow (2003–2006) and our study focused on the type of evidence cited by each guideline rather than by the specific recommendations made.

Despite a number of strengths, our study has some limitations. First, we did not systematically search for different guidelines, but instead examined only a small sample of guidelines; future studies should expand our work to explore the quality of evidence underlying guidelines in other topic areas. For example, we believe a systematic examination of all guidelines produced by a particular organization (or, alternatively, all published guidelines in a particular topic area) would provide useful additional insights. To do so, we advocate the use of explicit grading schemes such as those of CHEP or GRADE (as in the recently reported framework for World Health Organization Rapid Advice Guidelines [38]). However, while we found a high degree of inter-rater reliability for assessing whether RCT evidence was high quality or not (kappa 0.78 after all investigators completed a training set), future studies should also assess the inter-rater reliability scores for the GRADE or CHEP schemes if used by investigators less familiar with the schemes. Second, because the guidelines were inconsistent in how they cited studies in support of therapy recommendations (with some providing the citation directly with the recommendation and others providing numerous citations at the end of supporting text associated with recommendations), there is a potential risk we may have misattributed citations to particular recommendations. We attempted to minimize this risk by having two investigators extract recommendations and citations independently for each guideline and by always biasing in favor of the guideline (i.e., if several citations were attached to a recommendation, we assigned the highest evidence rating achieved by any of the studies to that recommendation). However, future researchers may want to consider prospectively surveying guideline developers to determine exactly which pieces of evidence are considered for each recommendation; also, documentation of the debate around the evidence for particular recommendations in different overlapping guidelines would provide potentially interesting insights. Finally, although our choice to restrict our analysis to cardiovascular therapy recommendations may be perceived as a limitation, it in fact strengthens our conclusions since therapy recommendations are those most likely to be based on RCT evidence. Thus, our findings represent a “best-case” scenario, insofar as very few preventive or diagnostic guideline recommendations are based on RCT evidence.

In conclusion, our finding that less than one-third of treatment recommendations (and less than half of those citing RCTs in support of the advocated treatment) were based on high-quality evidence in national evidence-based guidelines for common conditions should sound a note of caution to consumers of clinical practice guidelines who assume that the sobriquet “evidence based” means that all recommendations contained therein are derived from high-quality evidence. In particular, we have documented that even evidence arising from internally valid RCTs may not be directly applicable to the populations, interventions, and outcomes specified in a guideline recommendation. As a recent editorial noted, “external validity is the neglected dimension in evidence ranking” [39]. Indeed, in order to make the evidence base underlying therapy recommendations more transparent in future guidelines, we advocate wider adoption of evidence-rating schemes (such as the CHEP system or the GRADE system) that go beyond just judging the internal validity of supporting evidence but also incorporate considerations of the clinical relevance and applicability of that evidence to the clinical scenario the recommendation is being made for. A clearer understanding of the strengths and limitations of the underlying evidence base will then permit clinicians to individualize the application of practice guideline recommendations to their patients.

## Abbreviations

AGREE: Appraisal of Guidelines Research and Evaluation
CHEP: Canadian Hypertension Education Program
GRADE: Grading of Recommendations Assessment, Development and Evaluation
RCT: randomized controlled trial
